# Influence of Coffee upon the Sulphate of Quinine

**Published:** 1847-10

**Authors:** 


8. *Influence of Coffee upon the Sulphate of Quinine*.—According to the experiments of M. Dorvault, the sulphate of quinine, with the exception of a very small quantity, remains unchanged by the action of coffee. According to him, the disappearance of the bitter taste is due partly to the transformation of the portion of quinine which is dissolved into a tannate, and partly to the action of the other principles of coffee. M. D. thinks that it is only the dissolved portion of the sulphate which affects the organ of taste, and that this is decomposed by the tannin of the coffee, whilst the undissolved portions of the sulphate of quinine remain unchanged.

Sulphate of quinine dissolved by the aid of sulphuric acid or alcohol, looses but very little of its bitterness by admixture with coffee. Experience appears to have established the fact that the medicinal properties of the sulphate of quinine are not impaired by the action of coffee.

M. D. recommends the following formula for the administration of "*quininized coffee*,"

| | |
|---|---|
| R. Coffee, parched and ground, | 10 parts. |
| Boiling water, | 100 " |

Treat by displacement, filter and add sulphate of quinine 1 part and sugar 15 parts.—Translated from Bul. Gén. de Thèrap., April, 1847, for *Ibid*.

